# Alternating Hemiplegia of Childhood: neurological comorbidities and intrafamilial variability

**DOI:** 10.1186/s13052-021-01194-2

**Published:** 2022-02-17

**Authors:** Piero Pavone, Xena Giada Pappalardo, Naira Mustafa, Sung Yoon Cho, Dong Kyu Jin, Gemma Incorpora, Raffaele Falsaperla, Simona Domenica Marino, Giovanni Corsello, Enrico Parano, Martino Ruggieri

**Affiliations:** 1 Pediatric Clinic, Department of Clinical and Experimental Medicine, University Hospital A.U.O. “Policlinico-Vittorio Emanuele” of Catania, Catania, Italy; 2 National Council of Research, Institute for Research and Biomedical Innovation (IRIB), Unit of Catania, Catania, Italy; 3 Department of Biomedical and Biotechnological Sciences (BIOMETEC), University of Catania, Catania, Italy; 4 Department of Paediatrics, School of Clinical Medicine, University of Cambridge, Cambridge, UK; 5 Department of Clinical and Chemical Pathology, Faculty of Medicine, Cairo University, Cairo, Egypt; 6 Department of Pediatrics, Samsung Medical Center, Sungkyunkwan University School of Medicine, Seoul, South Korea; 7 Unit of Pediatrics and Neonatal Intensive Therapy, Department of Promotion of Maternal and Infantile and Internal Medicine Health, and Specialist Excellence “G. D’Alessandro”, University of Palermo, Palermo, Italy; 8 Unit of Pediatrics, Neonatology and Neonatal Intensive Care, and Pediatric Emergency, AOU “Policlinico”, PO “San Marco”, University of Catania, Catania, Italy; 9 Mother and Child Department, Operative Unit of Pediatrics and Neonatal Intensive Therapy, University of Palermo, Palermo, Italy; 10 Unit of Rare Diseases of the Nervous System in Childhood, Department of Clinical and Experimental Medicine, Section of Pediatrics and Child Neuropsychiatry, University of Catania, AOU “Policlinico”, PO “G. Rodolico”, Catania, Italy

**Keywords:** Alternating hemiplegia of childhood (AHC), Epilepsy, Comorbidities, *GRIN2A*, Case report

## Abstract

**Background:**

Alternating of Childhood (AHC) is an uncommon and complex disorder characterized by age of onset before 18 months with recurrent hemiplegia of one or either sides of the body or quadriplegia. The disorder is mainly caused by mutations in *ATP1A3* gene, and to a lesser extent in *ATP1A2* gene. In AHC neurological co-morbidities are various and frequently reported including developmental delay, epilepsy, tonic or dystonic spells, nystagmus,autonomic manifestations with intrafamilial variability.

**Case presentation:**

Clinical and genetic findings of a couple of twins (Family 1: Case 1 and Case 2) and a couple of siblings (Family 2: Case 3 and Case 4) coming from two different Italian families affected by AHC were deeply examined. In twins of Family 1, a pathogenic variant in *ATP1A3* gene (c.2318A>G) was detected. In siblings of Family 2, the younger brother showed a novel *GRIN2A* variant (c.3175 T > A), while the older carried the same *GRIN2A* variant, and two missense mutations in *SCNIB* (c.632 > A) and *KCNQ2* (1870 G > A) genes. Clinical manifestations of the four affected children were reported along with cases of AHC drawn from the literature.

**Conclusions:**

Hemiplegic episode is only a sign even if the most remarkable of several and various neurological comorbidities in AHC affected individuals. Molecular analysis of the families here reported showed that clinical features of AHC may be also the result of an unexpected genetic heterogeneity.

**Supplementary Information:**

The online version contains supplementary material available at 10.1186/s13052-021-01194-2.

## Introduction

Alternating Hemiplegia of Childhood (AHC) is an uncommon complex disorder, mainly characterized by paroxysmal episodes of repeated, transient paresis involving either or both sides of the body with onset usually before the age of 18 months. This disorder was first described in late 1960s by Verret and Steele on their study, which included eight children with migraine, among whom three showed the characteristic features of AHC [1]. Diagnostic criteria for AHC were expressed by Krageloh and Aicardi [2] and Bourgeois et al. [3] and consist of: a) onset before the age of 18-months; b) autonomic phenomena; c) cognitive impairment; d) repeated episodes of hemiplegia that sometimes involve both sides of the body; e) neurological abnormalities such as choreoathetosis; f) disappearance of the symptoms with the sleep and their resume after waking. The clinical signs of AHC are complex, heterogeneous, and follow a unique pattern: their clinical progression tends to occur in sequential distinctive phases, the paroxysmal episodes are often preceded by precipitating factors such as environmental stress, bathing and other events [4]. Non-paroxysmal features of subjects with AHC are various and include developmental delay/intellectual disability (DD/ID), epilepsy, autonomic dysfunctions, abnormal eye involvement, movement disorders, ataxia, dystonia, and choreoathetosis [4–8].

Although the pathophysiologic mechanism causing the clinical expression of the disorder remain unknown, significant research advances over the years, particularly those seen in the clinical genetics field, allowed a better mechanistic understanding of the attributed genes and enabled early diagnosis and precocious treatment. Relevant etiopathogenetic role in AHC is linked to mutations in *ATP1A2* (AHC-1; OMIM#104290) and in *ATP1A3* genes (AHC-2; OMIM#614820), respectively which encode two different alpha subunits of the Na^+^/K^+^ ATPase transmembrane ion pump [9, 10]. *ATP1A3* is by far more common than *ATP1A2* mutation [11]. It is worth mentioning that clinical expression of *ATP1A3* mutations are expanding suggesting the term *ATP1A3*-related disorder in complex cases [7].

Herewith, the main neurological manifestations occurred in a couple of twins (Family 1: Case 1 and Case 2) and a couple of siblings (Family 2: Case 3 and Case 4) affected by AHC and a revision of the cases of the literature were reported. The intrafamilial clinical variability as regards to the level of DD/ID, the frequency and intensity of hemiplegic episodes, seizures, and the presence of other co-morbidities observed in the present children were discussed. The mutational analysis of the AHC was performed in both couples of probands. A standard AHC diagnosis was confirmed by the *ATPA3* gene variant in the twins (Family 1: Case 1 and Case 2) but, not in siblings (Family 2) that carried a variant in *GRIN2A* gene in the younger brother (Case 3) and an unreported variants for AHC in *GRIN2A*, *SCN1B*, and *KCNQ2* genes in the older brother (Case 4). The genetic role of these variants in the clinical expression of AHC was discussed.

## Cases-report

### Family 1

#### Twin pair (case 1 and case 2)

Twin girls were born to unrelated Italian parents. The clinical features and course of disorder was rather similar in both girls but one (Case 2) exhibited milder manifestations as regards the frequency and the intensity of the AHC episodes. In both twins, ID was mild. A wider clinical manifestation of the twins has been previously reported [12]. The clinical onset started in their first few months of life with bath-induced paroxysmal events lasted 2 years. At 2 years, both twins had an episode of acute encephalopathy rapidly solved. Around 3 years, they presented with typical recurrent episodes of hemiplegia prevailing in the right side. Signs of distonic movements were further noted. In the following years, a marked reduction of hemiplegic attacks to the disappearance was observed with mild persistence of the other disturbances. During the adolescence, in both girls ID remained mild, instead brief dystonic movements and severe episodes of headache were registered.

### Family 2

#### Consent for publication

Written informed consent for publication of their clinical details was obtained from the patient’s parents. A copy of the consent form is available for review by the Editor of this journal.

#### Siblings pair

The siblings’ mother had atresia of the proximal part of the bile ducts and was admitted for liver transplantation in the first months of her life. The parents are neurologically normal and denied to have suffered from paralysis or seizures.

#### Case 3

**Fig. 1:**
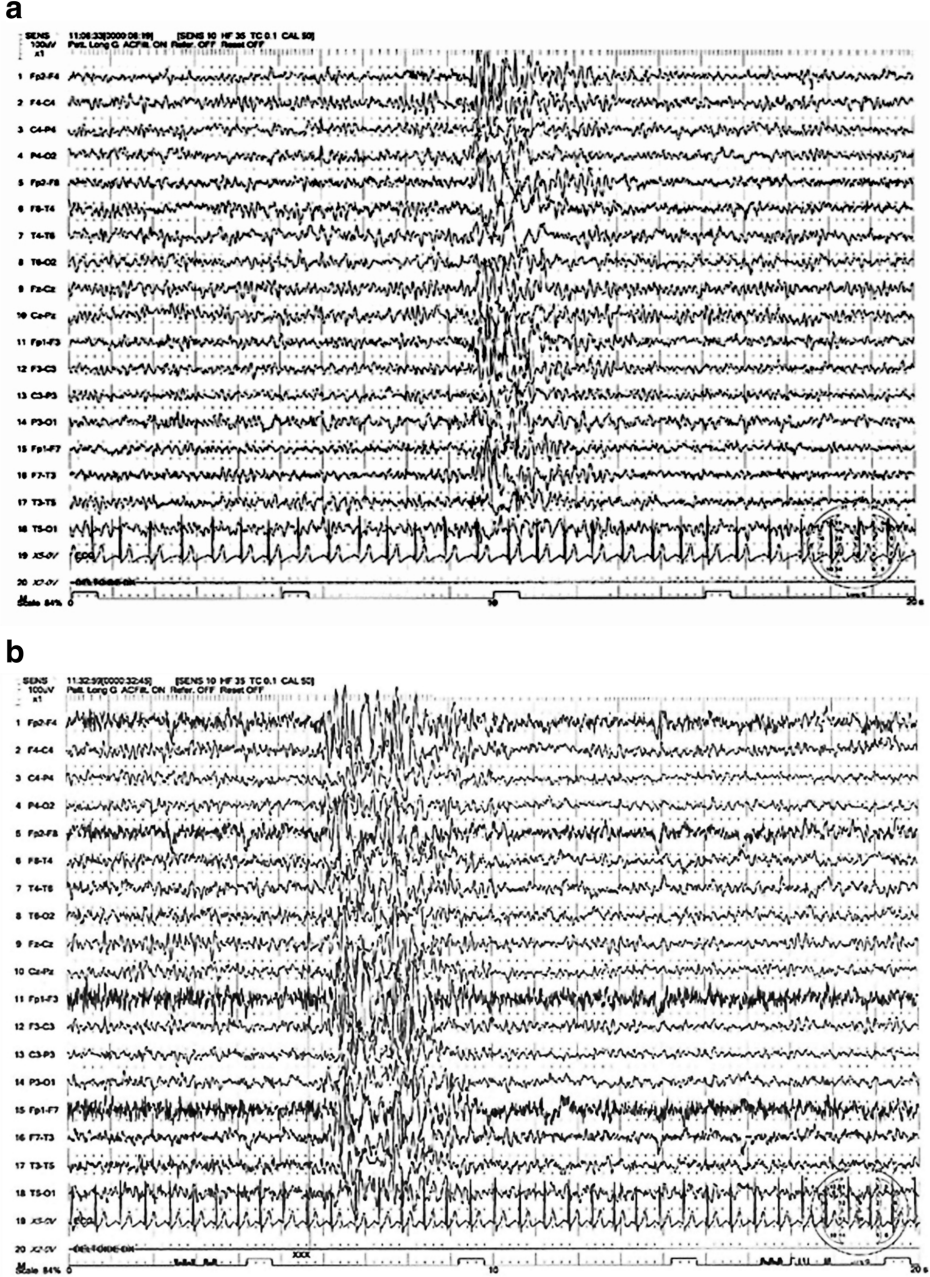
**a** Case 3. EEG at 4 years old showing multifocal spike/waves prevalently in fronto-centro-parietal regions. **b** Case 3. EEG at 4 years old showing multifocal spike/waves with generalization

This is a six and half years old male patient. He was born at the 37th week of gestation by cesarean section. The mother complained that the child was suffering from frequent episodes of upper airways infections and nocturnal cyanotic crises with breathing cessation due to obstructive sleep apnea syndrome (OSAS). The child grew up normally without specific clinical manifestations (except for episodes of OSAS) until 4 years old, when he experienced, for the first time, loss of bladder control followed by recurrent attacks of generalized tonic-clonic seizures in clusters. The video-EEG showed multifocal spike/wave pattern mainly evident in the Fronto-Centro-Parietal regions (Fig. 1 a-b). The child started treatment with valproate (20 mg/Kg/die), which managed to control the seizures. Few months later, he presented the first episode of AHC prevailing in right side, which was followed by inability to walk for 2 months. During this period, the child was fed by nasogastric tube because he was unable to swallow. During the following 2 years, he was admitted to Pediatric Clinic Institution (University of Catania, Italy) for several times in coincidence with the admission of his brother (Case 4, see next paragraph). He manifested frequent episodes of hemiplegia affecting prevalently the right side. The crises were less frequent and less severe compared to those of his brother. He attended the school with sufficient performance. At the most recent follow-up visit (seven and half years old), neurological examination displayed mild ID (IQ 64 WISC III), normal speech and language skills and notably reduced sensitivity to pain. The paroxysmal episodes of hemiparesis were infrequent (1–2 every 3 months) and no seizure was reported. Brain MRI, EMG, and NCS were normal. EEG in the awake state and during sleep showed same previous EEG anomalies with multifocal spikes and waves. Treatment consisted of valproate (20 mg/Kg/die) and levetiracetam (10 mg/Kg/die).

#### Case 4

**Fig. 2:**
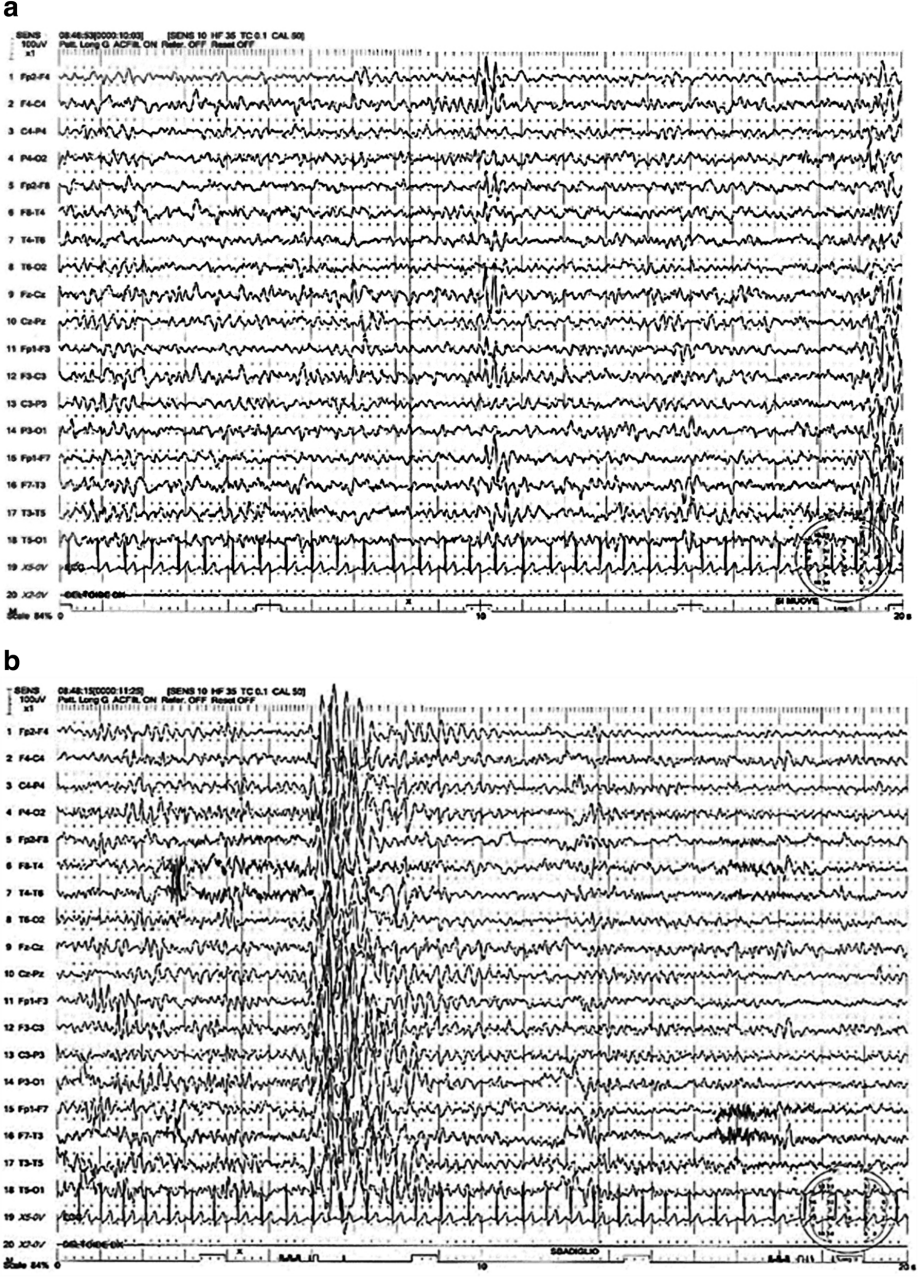
**a** Case 4. EEG at 8 years old showing multifocal spike/waves prevalently in the frontal regions. **b** Case 4. Ictal EEG showing multifocal spikes and waves

This is older brother who came to Pediatric Clinic Institution in Catania (Italy) at the age of 3 months because of seizures. He was born at the 39th week of gestation by cesarean section due to the threatened abortion and internal podalic version. At admission, trans-fontanellar ultrasound and EEG recordings were normal as well routine laboratory analysis. The child was discharged with valproate treatment at the dosage of 20 mg/Kg/die. In the subsequent months, two other seizures were recorded. At 18 months, he showed difficulty in standing up without support. The delay also involved the language development. Furthermore, the boy manifested an episode of generalized hypertonia of short duration followed by right hemiparesis resolved after 48 h initially referred to as Todd’s paralysis, but the clinical suspect of alternating hemiplegia of childhood (AHC) came out. At 2 years, the hemiplegic attacks were frequent as well as tonic and dystonic attacks. The child showed to be apathetic with poor social skills. At 2 years and 9 months, the boy continued to present unfrequent epileptic seizures (1–2 for months) no coincidentally occuring with episodes of hemiparesis mainly right sided. Treatment with flunarizine (10mg/Kg/die) was started, but irregularly conducted. At examination, after the hemiparetic episode, the affected side of the body was hypotonic, there was difficulty to stimulate patellar reflexes, and reduction of tactile, thermal, and pain sensibility were also noted. Some months later, the clinical manifestations of AHC involved alternatively both sides, and sometimes both sides at the same time. Video-EEG at awake and during the sleep showed multifocal spike and waves mainly expressed in the frontal region. Treatment with valproate was maintained 20 mg/Kg/die plus levetiracetam (10 mg/Kg/die) in add-on. During the following years, the epileptic seizures and paretic episodes were frequent and barely responsive to any type of treatment including flunarizine (10mg/Kg/die), irregularly conducted, and anticonvulsants. The hemiplegic manifestations showed a wide variability with some episodes lasting for one hour, and others being prolonged to several days up to 2–3 months. Serial EEG in the awake state and during the sleep showed the presence of multiple spike and wave mainly expressed in frontal region (a seizure recorded during the EEG registration) (Fig. 2 a-b). At 10 years old, the child showed a fairly good condition at physical examination. He attended the regular school with sufficient scholastic performance. At neurological examination, pain sensitivity was found to be reduced. Brain MRI was normal. At the most recent follow-up visit (11 years old), the parents referred that he attends the school regularly with support. He had a mild ID (IQ = 58) WISC III and mild speech impairment consisting of poor expressive vocabulary with scant sentences. He had very few clinical manifestations, and the attacks of hemiparesis and seizures became less frequent and less severe compared to the past. He is still taking valproate and levetiracetam at the same dosage as previously.

#### Mutation analysis

Total genomic DNA was extracted from peripheral blood samples for the mutational analysis, following informed consent by parents of Family 1 and 2 included in the present research.

For twins of Family 1, the molecular diagnosis was carried on *ATP1A2* and *ATP1A3* genes by sequencing of PCR-amplified DNA. PCR products amplified by LightCycler 480® (Roche Life Science) were determined by direct sequencing on ABI Prism® 3100 Genetic Analyzer (Thermo fisher Scientific) according to the manufacturer’s protocol. Sequence analysis was done by Applied Biosystems DNA Sequencing Analysis v.5.1 and ABI PRISM 310 Data Collection Software Version 3.1 software (ThermoFisher Scientific). Analysis of the pathogenic variant was performed using public repositories of genome variation, such as dbSNP and ClinVar database.

For siblings of Family 2 and their parents whole exome sequencing (WES) was carried. WES analysis was performed using the Illumina TruSight One panel. The samples were sequenced by using the Illumina NextSeq 500 platform (Illumina Inc.) with 2 × 150 bp paired-end reads. Alignments and variant calls were generated using NextGene software (v2.4.1, 2015). Variant calls (with coverage <15X) were limited to the genes of interest. For the clinical interpretation of genomic variants was used Alamut-Batch (Version 1.4.0, 2015), the high-throughput annotation software for NGS analysis. Alamut Visual (Version 2.7) was used for integrating genetic and predictive information on missense, nonsense, frameshift, and splice-site variants, providing computational algorithms for SIFT, PolyPhen-2 (Version 2.2.2, 2012) and Mutation taster. Variants were annotated for minor allele frequencies in the Exome Aggregation Consortium (ExAC) database (Version 0.3), and heterozygous variants with minor allele frequencies > 0.01 (1%) were filtered out. Variants were classified as pathogenic/likely pathogenic/VUS/likely benign/benign according to the 2015 American College of Medical Genetics and Genomics (ACMG) guidelines [13]. The validation of variants was performed using Sanger sequencing in the probands.

## Results

The candidate gene approach identified in twin girls a pathogenic variant p.Asn773Ser of *ATP1A3* gene (rs606231437) associated with AHC-2 (Table 2) [12]. In the siblings, WES analysis found no mutations in *ATP1A3* and *ATP1A2* genes, identifiying three potentially pathogenic missense variants of *GRIN2A*, *SCN1B* and *KCNQ2* genes in the older brother (as in the asymptomatic father) (Table 1), while in the youngest brother a *GRIN2A* variant was detected (as in the healthy father). The missense substitution c.3175 T > A (p. Ser1059Thr) in *GRIN2A* (*Glutamate Ionotropic Receptor NMDA Type Subunit 2A*) gene is the only shared between siblings and their father and it has not been previously reported in other genomic variation databases. Two missense mutations, c.632G > A (p.Cys211Tyr) in *SCN1B (Sodium Voltage-Gated Channel Beta Subunit 1)* gene and c.1870G > A (p.Gly624Arg) in *KCNQ2* (*Potassium Voltage-Gated Channel Subfamily Q Member 2*) gene both found in the firstborn and the healthy father are listed respectively as rs150721582 and rs771211103 in gnomAD, 1000 Genome project, ClinVar, and dbSNP database. The pathogeniticy prediction done with the MutationTaster tool shows that all of three variants may have a deleterious impact on the function and structure of the protein since the wildtype amino acid position is highly conserved across vertebrate species, and the effects of these changes might also determine potential splice site variants.

**Table 1:** Mutational results of Family 1 (Case 1 and Case 2) and Family 2 (Case 3 and Case 4)

			**Family 1**			
**Gene**	**Variant**	**AA change**	**Twin 1**	**Twin 2**	**F**	**M**
*ATP1A3* (NM_1522969)	c.2318A > G	p.Asn773Ser	AG	AG	N/A	N/A
			**Family 2**			
**Gene**	**Variant**	**AA change**	**Brother 1**	**Brother 2**	**F**	**M**
*GRIN2A* (NM_001134408)	c.3175 T > A	p.Ser1059Thr	TA	TA	TA	N/D
*SCN1B* (NM_001037)	c.632G > A	p.Cys211Tyr	GA	N/D	GA	N/D
*KCNQ2* (NM_172107)	c.1870G > A	p.Gly624Arg	GA	N/D	GA	N/D

*Abbreviations*: *F* father, *M* mother, *AA* aminoacid, *Heterozygous* AG (family 1), *TA* GA (family 2), *N/A* not available, *N/D* not detected

## Discussion

The four children here reported, twin girls and two male siblings, are representative example of typical AHC disorder, all presenting clinical manifestations, which fall within the diagnostic criteria for the disorder [2, 3]. A pathogenetic variant p.Asn773Ser of *ATP1A3* gene (rs606231437) was reported in the twins (Family 1: Case 1 and Case 2). As regards the siblings (Family 2: Case 3 and Case 4), the younger child (Case 3) showed a novel *GRIN2A* variant *(*p. Ser1059Thr) reported also in his healthy father, while *GRIN2A* variant plus *SCN1B* (p.Cys211Tyr; rs150721582) and *KCNQ2* (p.Gly624Arg; rs771211103) variants were found in the older brother (Case 4) and paternally inherited too.

Mikati et al. have demonstrated that clinical course of patients with AHC is complex and evolves in three distinct phases [4]. Phase one begins during the first few months of life and continues for 1 year and in this phase the most common features consist of unilateral nystagmus, ocular deviation, dystonic spells, and developmental delay. Phase two lasts from the age of one to 5 years, in which the hemiplegic spells become more typical, with a possible frequency of several times each month, and with a duration of several days or even weeks. In this phase, abnormal movements, dystonic attacks, and choreoathetosis are frequently observed. Phase three is represented by fixed neurologic deficits and obvious ID. In this phase, dystonic and hemiplegic episodes become less frequent and less severe. Main clinical manifestations of the four probands compared to those indicated by Mikati et al. [4] and were summarized in Table 2. Identification of AHC pattern and how the symptoms progress may facilitate earlier diagnosis of this disorder bearing in mind that the symptoms are wide particularly regarding the duration and frequency of hemiplegic and dystonic episodes. The beneficial effect of sleep on abnormal paroxysmal features with the disappearance of paroxysmal phenomena and resumption of the normal movements is one of the diagnostic criteria of AHC. Remission of the symptoms may be observed even after a short nap. A study on sleep architecture was carried out in four AHC children and the results showed a normality on the sleep structure, sleep duration, cycle length, rapid eye movement (REM) latency, and REM and slow wave sleep (SWS) percentages [14]. Clinical suspicion starts when the infant presents with abnormal ocular movements such as nystagmus and ocular deviation, head deviation, dystonic spells and unilateral hypotonia, which are usually triggered by several factors including light, sound, exposure to heat or cold, and stress whether physical or psychological. Paroxysmal hemiplegic episodes usually start after the first year of life and usually fluctuate from a side to the other or occur simultaneously on both sides. These episodes may be accompanied by speech impairment, gait incoordination, and movement disorders. The first diagnostic approach is to exclude a diagnosis of epilepsy, which can precede, co-occur with, or follow the hemiplegic attacks. A prolonged Video EEG is pivotal for differentiating seizures from the paroxysmal events of AHC. Additional standard diagnostic tests are listed in supplementary file (S1). Concerning the severity of the condition, AHC is usually reported as devastating since hemiplegic features are often associated to other neurologic dysfunctions including severe DD/ID and epileptic seizures, as discussed in the following paragraphs.

**Table 2:** Course of clinical manifestations of Family 1 (Case 1 and Case 2), Family 2 (Case 3 and Case 4) and AHC phases reported by Mikati et al. (2000)

	Family 1										Family 2						Mikati et al. (2000)		
	Case 1					Case 2					Case 3			Case 4			AHC phases		
**Age**	**0–24 mo**	**24–28 mo**	**3–7 y**	**7–11 y**	**11–19 y**	**0–24 mo**	**24–28 mo**	**3–7 y**	**7–11 y**	**11–19 y**	**0–1 y**	**1–5 y**	**6–7 y**	**0–1 y**	**1–5 y**	**6–11 y**	**Phase 1**	**Phase 2**	**Phase 3**
**Features**																	**(0–1 y)**	**(2–5 y)**	**(+5y)**
**Dystonic induced events**	+++	+	–	–	–	++	+	–	–	–	–	–	–	–	–	–	–	–	–
**Abnormal ocular movements**	+++	+	–	–	–	++	+	–	–	–	–	–	–	–	–	–	+	+++	–
**DD/ID**	+/−	+/−	+/−	+/−	+/−	+/−	+/−	+/−	+/−	+/−	+/−	+/−	+/−	–	+/−	+/−	+	+++	+++
**Autonomic phenomena**	++	++	–	–	–	+	+	–	–	–	–	++	+	–	+	+	+	++	–
**Hemiplegic attacks**	+	++	+++	++	+	+	+	++	+	–	–	++	+	–	+++	+/−	+	+++	++
**Tonic/dystonic attacks**	+	++	+++	++	+	+	+	++	+	+/−	–	++	+	–	+++	+	+	+++	++
**Acute encephalopathy**	–	++	–	–	–	++	–	–	–	–	–	–	–	–	–	–	–	–	–
**Epileptic seizures**	–	–	–	–	–	–	–	–	–	–	–	++	–	+	+++	+	–	+	+
**Headache with aura**	–	–	–	+	+++	–	–	–	+	++	–	–	–	–	–	–	–	–	–
**Walking problems**	+	+	+	+	+	+	+	+	+	+	–	+	–	–	–	+	–	–	–

*Abbreviations*: Level of severity are indicated from +/− to +++, *DD/ID* Developmental Delay/Intellectual Disability

### Developmental delay and cognitive impairment

Mikati et al. [4] reported that developmental delay was observed in 40 out of 44 patients enrolled in their study. According to these authors, developmental level correlates with the age of AHC individuals and with the age of onset of the hemiplegic episodes. Although neuropsychological evaluation showed wide variability in functional impairment for cognitive, adaptive and behavioral domains, younger patients demonstrated better results. It remains to establish whether the cognitive delay in AHC individuals is related to the repeated attacks of hemiplegia or to a primary effect of the disorder [4]. In the study of Sweney et al. [8], cognitive impairment was generally defined by the parents as mild to moderate, and recently a mild cognitive impairment form was reported by Polanowska et al. [15] in two adult patients, in whom a neuropsychological examination showed a normal or near normal global cognitive functioning, with dificits only in some isolated executive functions. In the children here reported the ID was mild and without a progressive course.

### Epilepsy

Epileptic seizures are reported in about 50% of AHC individuals (Table 3) [4, 8, 16–18]. In the study of Mikati et al. [4] only 8 (19%) out of 44 patients experienced epileptic seizures, which occurred infrequently in one-half of these patients (three seizures or less). Out of those eight patients, four presented with generalised tonic clonic seizures, three with focal clonic seizures, and one with generalised myoclonic seizures. Status epilepticus appeared only in one patient. According to Sweney et al. [8], 44 (43%) out 103 AHC individuals showed generalized tonic clonic seizures. The mean age of onset of seizures was around 6 years, with 10 (23%) of the 44 cases who did not experience epileptic episodes until the age of 10 years or later. Ictal EEG seizures were reported by Saito et al. [16] in AHC individuals. In another study, status epilepticus appeared in 4 out of 9 patients at the age of 6–16 years [17]. In a report of Uchitel et al. [18], on 51 patients with AHC, 32 (62.7%) had focal epilepsy in different cerebral regions, but more frequently frontal region; 11 (21.5%) showed primary generalized tonic clonic seizures, myoclonic seizures, and/or absences. In 8 (15.5%) patients, seizures preceded other AHC paroxysmal events. However, according to Heinzen et al. [10] seizures may precede the paroxysmal hemiplegic episodes and EEG registration may appear initially normal. In the present study, twins never complained of seizures up to the current age of 19 years, whereas epileptic seizures were recorded in siblings showing focal seizures with onset in Case 3 at 3 years and half, and in the other one at 4 years. In both siblings EEG showed multifocal spike and wave expressed mainly in the frontal region. In general, seizures are reported with low frequency and good response to treatment. A clinical distinction between episodes of hemiplegic attacks and epileptic seizures is not always clear, and the correlation between the epileptic and hemiplegic episodes remains doubtful [16]. Ictal episode was registered in one of the siblings here reported.

**Table 3:** Summary of epileptic seizures in AHC cases of the present study and from literature

Authors	No. Cases	Type of seizures
*Mikati* et al. *2000*	8/44 (19%)	4 GTCS; 3 FCS; 1 GMS
*Sweney* et al. *2009*	44/103 (43%)	44 GTCS
*Saito* et al. *2010*	1	ES
*Rosewich* et al. *2014*	4/9	4 SE
*Uchitel* et al. *2019*	51	32 (62%) FS (mainly frontal); 11 (21%) GTCS-MS-Absence; 8 ES
Present cases	2/4	1 FS/1 MFS

*Abbreviations*: *GMS* Generalized Myoclonic Seizures, *GTCS* Generalized Tonic-Clonic Seizures, *ES* Epileptic Seizures, *FCS* Focal Clonic Seizures, *FS* Focal Seizures, *MS* Myoclonic Seizures, *MFS* Multifocal seizures*SE* Status Epilepticus

### Migraine

Migraine is a symptom not commonly found in AHC neither in the affected individuals nor in the family history [8]. Howver, in the reported twins, the episodes of migraine with aura were one of most prevalent symptoms.

### AHC related disorders

Cognitive impairment, seizures, persistent movement disorders, and autonomic dysfunctions are considered comorbidities of the AHC. Nevertheless, after refining the symptoms of AHC by Krägeloh et al. [2] in 1980, and by Bourgeois et al. [3] in 1993, it became obvious that these symptoms may be recorded as primary components of AHC. *ATP1A3* has been implicated aside to AHC syndrome to other complex syndromes including the Rapid-onset Dystonia-Parkinsonism [6, 19, 20], and the Cerebellar ataxia, Areflexia, Pes cavus, Optic atrophy, and Sensoryneural hearing loss (CAPOS) syndrome [7, 21, 22]. At their current childhood/adolescent age, no one of the four cases here reported showed clinical features consistent with the uppermentioned syndromes.

#### Variability

Variability in clinical expression of paroxysmal and non-paroxysmal episodes in AHC individuals is well known. In the present cases, the intrafamilial variable clinical expression was observed as regard to the intensity and frequency of clinical features more pronounced in Case 1 between twins and in Case 3 between siblings. The cognitive impairment was mild in both twins and no seizures were recorded. In siblings, the seizures were more severe in Case 4 who showed more marked hemiplegic attacks. Cognitive impairment was mild in both siblings and speech delay was reported only in the oldest sibling. It is presumable that epigenetic events have conditioned the intrafamilial clinical variability.

#### Diagnosis

The diagnosis of AHC is mainly clinical but may be supported by molecular analysis. Typical gene mutations involved in the pathogenesis of AHC are located in *ATP1A2* and in *ATP1A3* genes as found in twins, but in some cases of AHC, however, these mutations are not found (as in children of Family 2). At WES analysis, we found in the Case 4 the identical haplotype inherited from the asymptomatic father, constituted of three heterozygous variants in *GRIN2A* (c.3175 T > A), *SCN1B* (c.632G > A) and *KCNQ2* (c.1870G > A) gene, while in the younger child (Case 3), who had a milder phenotype, only the variant in *GRIN2A* gene as in the healthy father was found. The *GRIN2A* gene encoding the NMDA receptor (NMDAR) subunit GluN2A has been suggested to constitute a locus for mutations in a subset of individuals with early-onset seizures [23]. Additionally two likely pathogenetic variants *SCN1B* (rs 150,721,582) and the *KCNQ2* (rs771211103) genes have been implicated in childhood epilepsies [24–26]. The role of three variants in the clinical expression observed in the children of family 2 is difficult to explain since the variants were also found in the healthy father and no previous cases of AHC with these variants have been reported.

#### Treatment

To date, no drugs are available to cure AHC. The treatment usually comprises multiple drug therapy regimen. The aim of these therapeutic agents is prophylactic against the paroxysmal attacks. Flunarizine is a calcium channel blocker that has been widely indicated as the most effective drug for AHC treatment [4, 6, 11, 27, 28]. The results achieved suggested that flunarizine therapy reduced the duration and severity of hemiplegic attacks, but did not interfere with the natural course of the disease. No severe side-effects have been seen in any patients during the time of treatment [17]. Recently, new treatment modalities have been proposed with triheptanoin [17], aripiprazole, [29] and verapamil [30] with notable reduction in frequency, severity, and duration of the hemiplegic attacks. In association to flunarizine, anticonvulsants have been applied in the treatment of the seizures using benzodiazepine, carbamazepine, barbiturates and valproate [31, 32]. In the siblings, valproate and leveticaretam managed to control the seizures.

## Conclusions

AHC is a complex, often serious disorder in which the hemiplegic episode is only one sign even if the most remarkable of several other body-system impairments involving autonomic nervous system, musculoskeletal system and brain with seizures and cognitive dysfunction as relevant clinical association. Clinical features and genetic analysis may result in prompt diagnosis and precocious treatment.

## Supplementary Information

**Additional file 1: S1. AHC diagnostic and laboratory test.** Routine laboratory examination, plasma amino acids, urine organic acids, blood lactate, pyruvate, urea, ammonia, thyroid functions, arterial blood gases (ABG), EEG, Video-EEG, MRI and MRI angiography are effective to exclude metabolic disorders and vascular diseases having the same pattern of features such as homocystinuria, organic acidurias (glutaric aciduria), urea cycle disorders (ornithine transcarbamylase deficiency, carbamoyl phosphate synthetase I deficiency, and citrullinemia) and Moyamoya disease. Diagnostic check-up may also include analysis of pterins, 5-methyltetrahydrofolate (5-MTHF) and monoamine metabolites in the cerebrospinal fluid.

## Data Availability

All data generated or analysed during this study are included in this published article.

## Abbreviations

ACMG: American College of Medical Genetics and Genomics
AHC: Alternating Hemiplegia of Childhood
ABS: Arterial Blood Gases
*ATP1A2*: ATPase Na+/K+ Transporting Subunit Alpha 2
*ATP1A3*: ATPase Na+/K+ Transporting Subunit Alpha 3
CAPOS: Cerebellar ataxia-areflexia-pes cavus-optic atrophy-sensorineural hearing loss
DD: Developmental Delay
EEG: Electroencephalogram
*GRIN2A*: Glutamate Ionotropic Receptor NMDA Type Subunit 2A
ID: Intellectual Disability
*KCNQ2*: Potassium Voltage-Gated Channel Subfamily Q Member 2
MRI: Magnetic Resonance Imaging
NMDAR: N-methyl-D-aspartate receptor
OSAS: Obstructive Sleep Apnea Syndrome
REM: Rapid Eye Movement
*SCN1B*: Sodium Voltage-Gated Channel Beta Subunit 1
SWS: Slow Wave Sleep
WES: Whole Exome Sequencing
