# Supplementary material for: Alternating Hemiplegia of Childhood: neurological comorbidities and intrafamilial variability
Source: Ital J Pediatr. 2022 Feb 17;48:29. doi: 10.1186/s13052-021-01194-2 (PMC8851838; doi:10.1186/s13052-021-01194-2)
Supplement: Supplementary file 1 — Additional file 1: S1. AHC diagnostic and laboratory test. Routine laboratory examination, plasma amino acids, urine organic acids, blood lactate, pyruvate, urea, ammonia, thyroid functions, arterial blood gases (ABG), EEG, Video-EEG, MRI and MRI angiography are effective to exclude metabolic disorders and vascular diseases having the same pattern of features such as homocystinuria, organic acidurias (glutaric aciduria), urea cycle disorders (ornithine transcarbamylase deficiency, carbamoyl phosphate synthetase I deficiency, and citrullinemia) and Moyamoya disease. Diagnostic check-up may also include analysis of pterins, 5-methyltetrahydrofolate (5-MTHF) and monoamine metabolites in the cerebrospinal fluid. [file 13052_2021_1194_MOESM1_ESM.docx]

# **Alternating Hemiplegia of Childhood, neurological comorbidities, intrafamilial variability. Case-report.**

**Piero Pavone^1^, Xena Giada Pappalardo^2,3^, Naira Mustafa^4,5^**, **Sung Yoon Cho^6^, Dong Kyu Jin^6^, Gemma Incorpora^1^, Raffaele Falsaperla^7^, Simona Domenica Marino^8^, Giovanni Corsello^9^, Enrico Parano^2^, Martino Ruggieri^10^**

^1^ Unit of Clinical Pediatrics, AOU "Policlinico", PO "G. Rodolico", University of Catania, Catania, Italy;

^2^ National Council of Research, Institute for Research and Biomedical Innovation (IRIB), Unit of Catania, Italy;

3 Department of Biomedical and Biotechnological Sciences (BIOMETEC), University of Catania, Italy;

^4^ Department of Paediatrics, School of Clinical Medicine, University of Cambridge, UK;

^5^ Department of Clinical and Chemical Pathology, Faculty of Medicine, Cairo University, Egypt;

*^6^* Department of Pediatrics, Samsung Medical Center, Sungkyunkwan University School of Medicine, Seoul, Korea;

7 Unit of Pediatrics and Neonatal Intensive Therapy, Department of Promotion of Maternal and Infantile and Internal Medicine Health, and Specialist Excellence "G. D'Alessandro", University of Palermo, Palermo, Italy;

^8^ Unit of Pediatrics, Neonatology and Neonatal Intensive Care, and Pediatric Emergency, AOU "Policlinico", PO "San Marco", University of Catania, Catania, Italy;

^9^ Mother and Child Department, Operative Unit of Pediatrics and Neonatal Intensive Therapy, University of Palermo, Palermo, Italy;

^10^ Unit of Rare Diseases of the Nervous System in Childhood, Department of Clinical and Experimental Medicine, Section of Pediatrics and Child Neuropsychiatry, University of Catania, AOU "Policlinico", PO "G. Rodolico".

**S1. AHC diagnostic and laboratory test.**

Routine laboratory examination, plasma amino acids, urine organic acids, blood lactate, pyruvate, urea, ammonia, thyroid functions, arterial blood gases (ABG), EEG , Video-EEG, MRI and MRI angiography are effective to exclude metabolic disorders and vascular diseases having the same pattern of features such as [homocystinuria](https://jamanetwork.com/journals/jamaneurology/fullarticle/799201#32619075), organic acidurias (glutaric aciduria), urea cycle disorders (ornithine transcarbamylase deficiency, carbamoyl phosphate synthetase I deficiency, and citrullinemia) and Moyamoya disease. Diagnostic check-up may also include analysis of pterins, 5-methyltetrahydrofolate (5-MTHF) and monoamine metabolites in the cerebrospinal fluid.


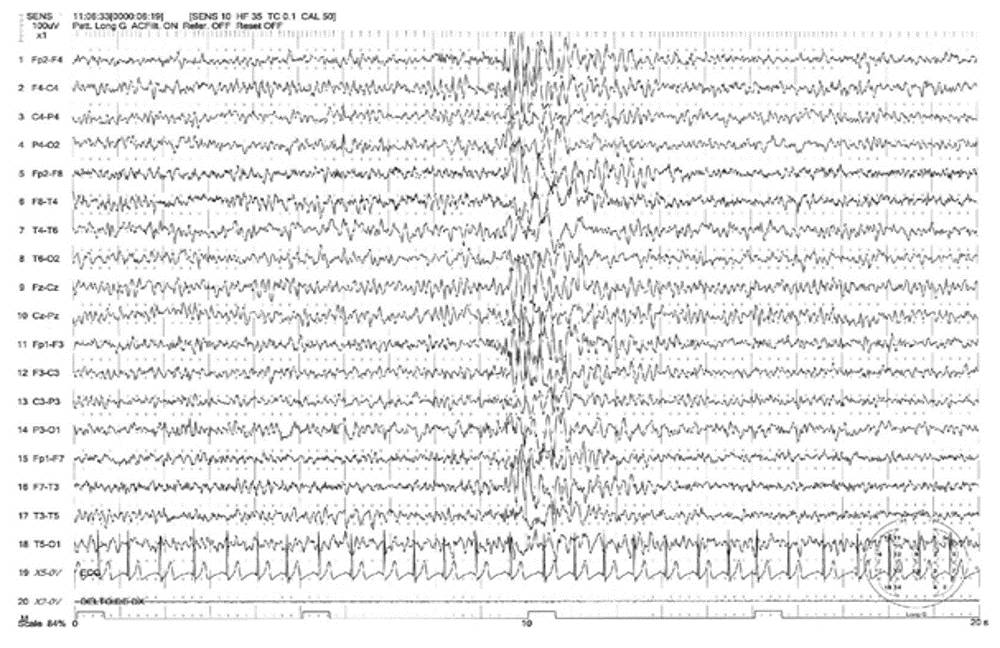


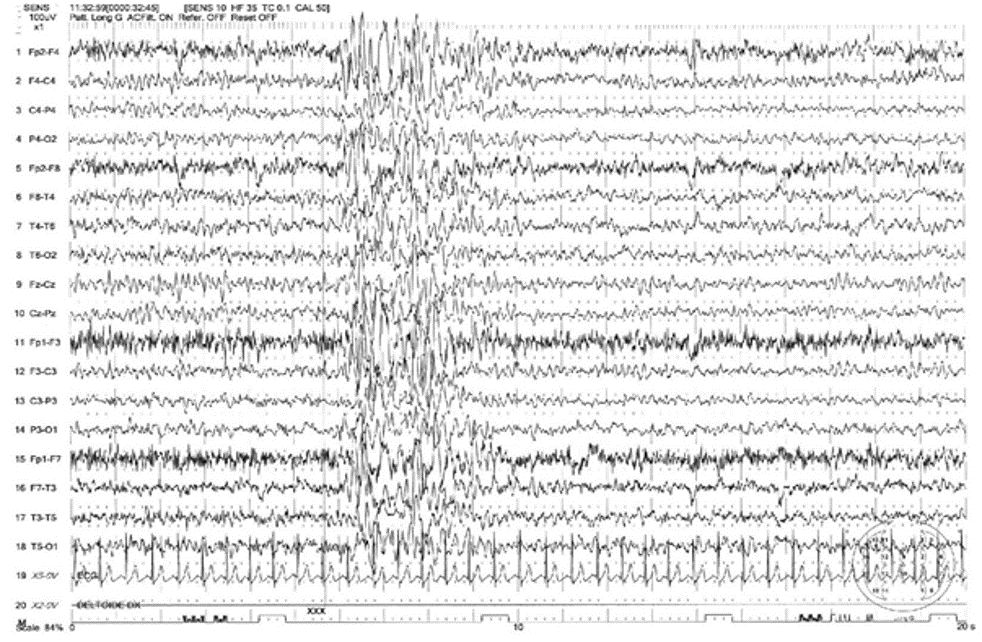


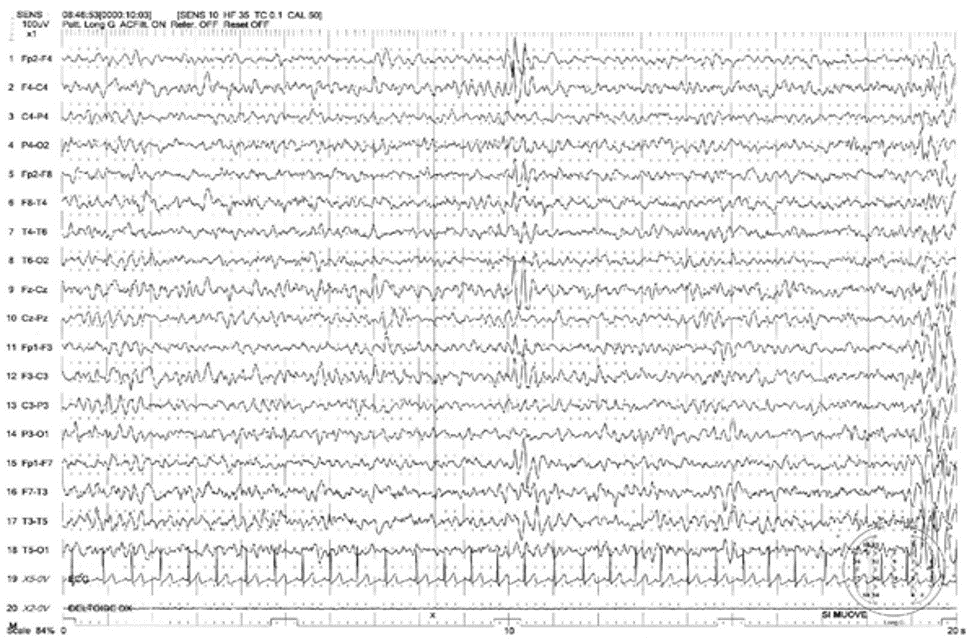


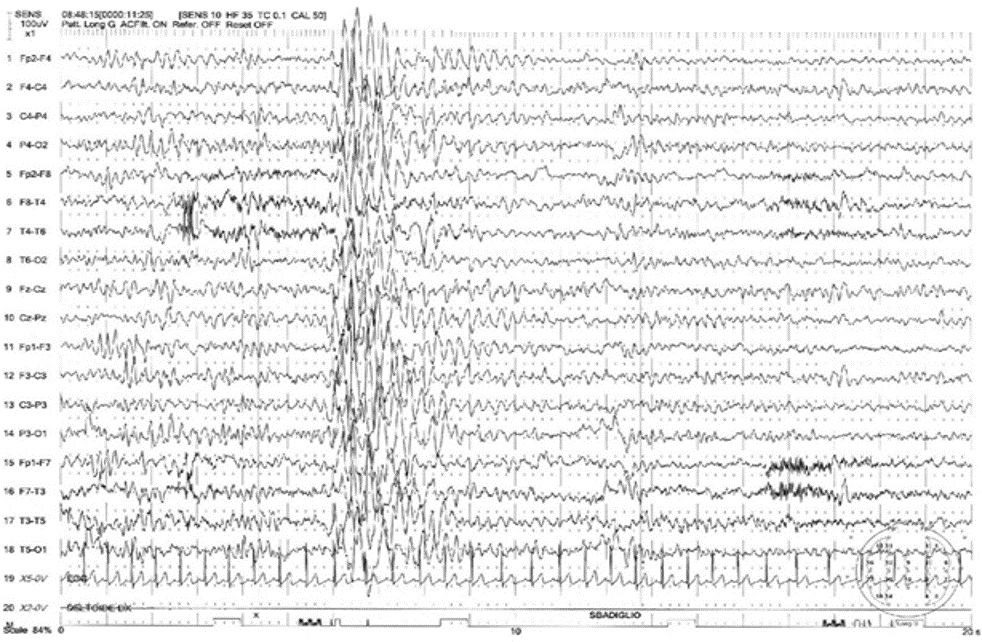


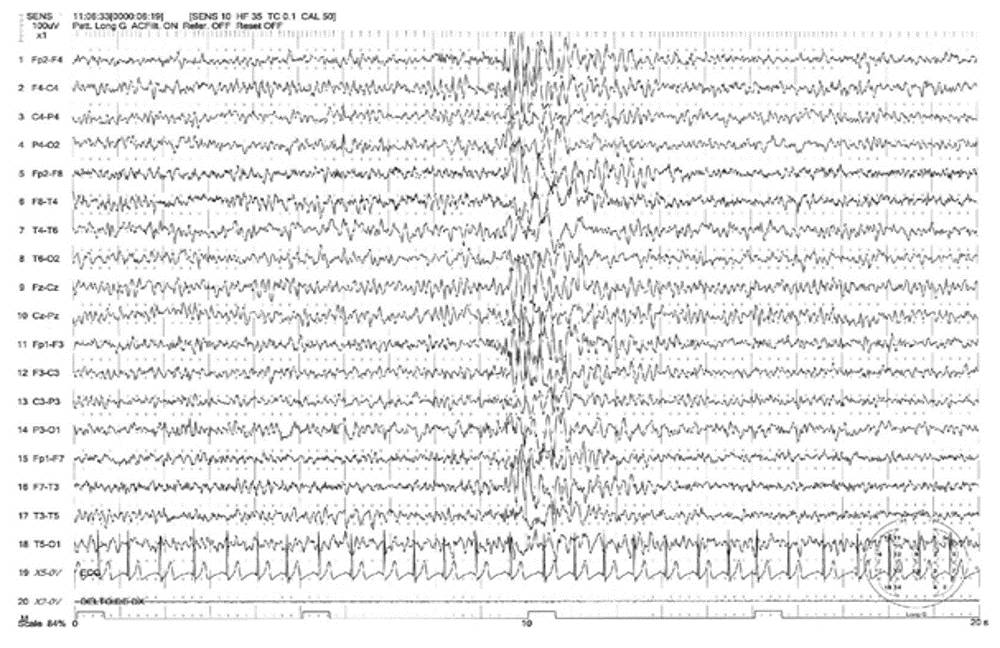


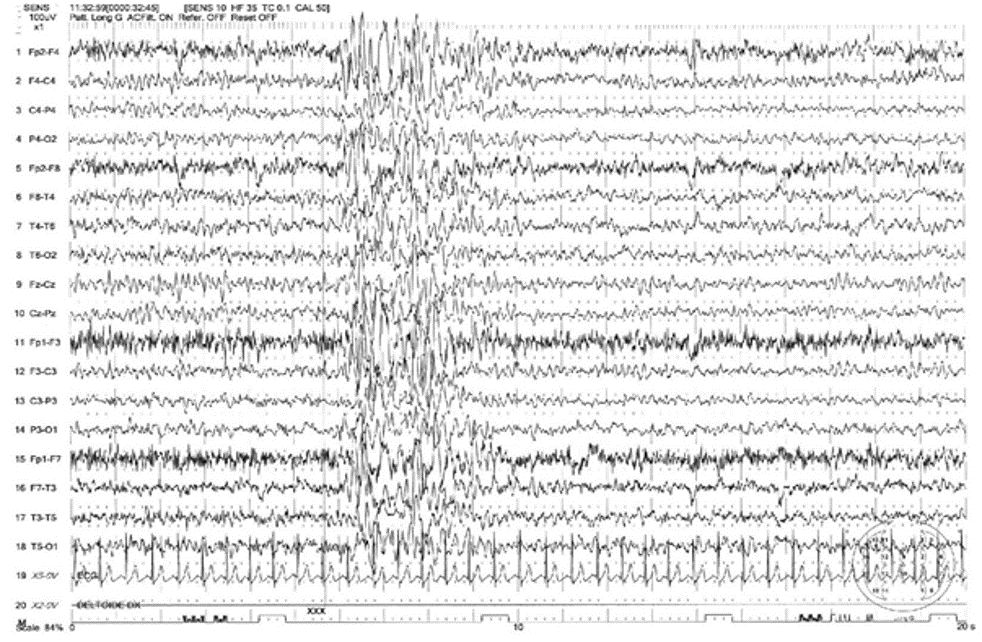


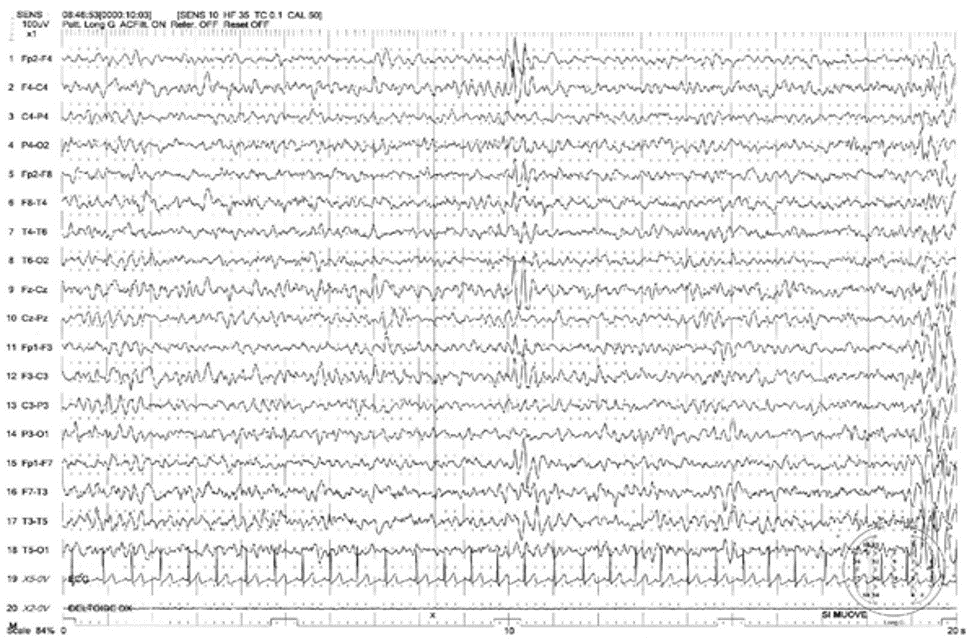


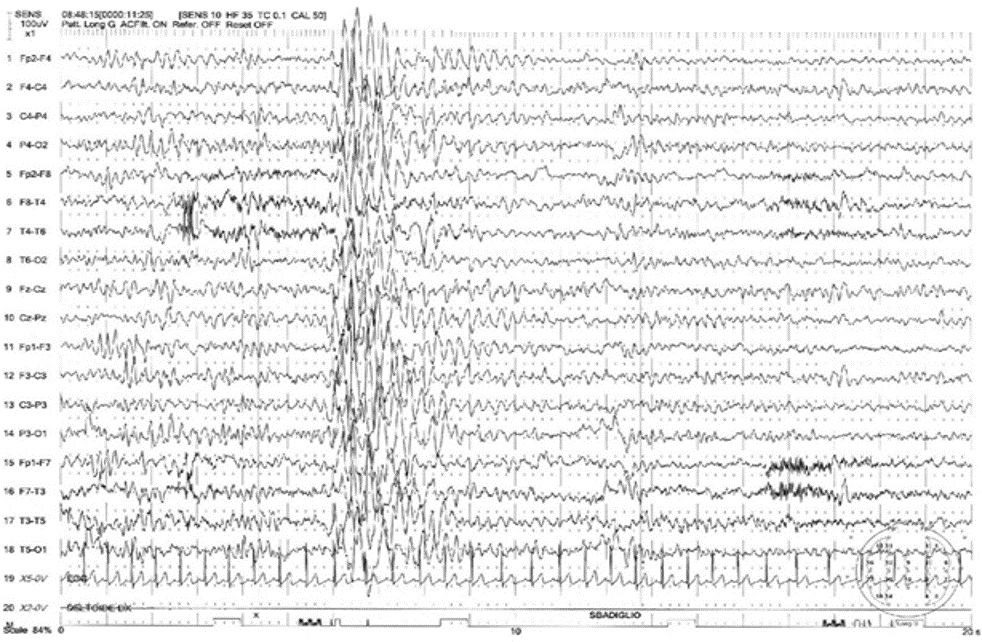


**Table 1. Mutational results of Family 1 (Case 1 and Case 2) and Family 2 (Case 3 and Case 4).**

|  | | | **Family 1** | | | |
| --- | --- | --- | --- | --- | --- | --- |
| **Gene** | **Variant** | **AA change** | **Twin 1** | **Twin 2** | **F** | **M** |
| *ATP1A3* (NM_1522969) | c.2318A>G | p.Asn773Ser | AG | AG | N/A | N/A |
|  | | | **Family 2** | | | |
| **Gene** | **Variant** | **AA change** | **Brother 1** | **Brother 2** | **F** | **M** |
| *GRIN2A* (NM_001134408) | c.3175T>A | p.Ser1059Thr | TA | TA | TA | N/D |
| *SCN1B* (NM_001037) | c.632G>A | p.Cys211Tyr | GA | N/D | GA | N/D |
| *KCNQ2* (NM_172107) | c.1870G>A | p.Gly624Arg | GA | N/D | GA | N/D |

*Abbreviations*: F=father; M=mother; AA= aminoacid; Heterozygous=AG (family 1); TA, GA (family 2); N/A= not available; N/D= not detected.

**Table 2 Course of clinical manifestations of Family 1 (Case 1 and Case 2), Family 2 (Case 3 and Case 4) and AHC Cases reported by Mikati et al. (2000).**

|  | **Family 1** | | | | | | | | | | **Family 2** | | | | | | **Mikati et al. (2000)** | | |
| --- | --- | --- | --- | --- | --- | --- | --- | --- | --- | --- | --- | --- | --- | --- | --- | --- | --- | --- | --- |
|  | **Case 1** | | | | | **Case 2** | | | | | **Case 3** | | | **Case 4** | | | **AHC phases** | | |
| **Age** | **0-24 mo** | **24-28 mo** | **3-7 y** | **7-11 y** | **11-19 y** | **0-24 mo** | **24-28 mo** | **3-7 y** | **7-11 y** | **11-19 y** | **0-1 y** | **1-5 y** | **6-7 y** | **0-1 y** | **1-5 y** | **6-11 y** | **Phase 1** | **Phase 2** | **Phase 3** |
| **Features** |  |  |  |  |  |  |  |  |  |  |  |  |  |  |  |  | **(0-1 y)** | **(2-5 y)** | **(+5y)** |
| **Dystonic induced events** | +++ | + | - | - | - | ++ | + | - | - | - | - | - | - | - | - | - | - | - | - |
| **Abnormal ocular movements** | +++ | + | - | - | - | ++ | + | - | - | - | - | - | - | - | - | - | + | +++ | - |
| **DD/ID** | +/- | +/- | +/- | +/- | +/- | +/- | +/- | +/- | +/- | +/- | +/- | +/- | +/- | - | +/- | +/- | + | +++ | +++ |
| **Autonomic phenomena** | ++ | ++ | - | - | - | + | + | - | - | - | - | ++ | + | - | + | + | + | ++ | - |
| **Hemiplegic attacks** | + | ++ | +++ | ++ | + | + | + | ++ | + | - | - | ++ | + | - | +++ | +/- | + | +++ | ++ |
| **Tonic/dystonic attacks** | + | ++ | +++ | ++ | + | + | + | ++ | + | +/- | - | ++ | + | - | +++ | + | + | +++ | ++ |
| **Acute encephalopathy** | - | ++ | - | - | - | ++ | - | - | - | - | - | - | - | - | - | - | - | - | - |
| **Epileptic seizures** | - | - | - | - | - | - | - | - | - | - | - | ++ | - | + | +++ | + | - | + | + |
| **Headache with aura** | - | - | - | + | +++ | - | - | - | + | ++ | - | - | - | - | - | - | - | - | - |
| **Walking problems** | + | + | + | + | + | + | + | + | + | + | - | + | - | - | - | + | - | - | - |

**Table 3 Summary of epileptic seizures in AHC cases of the present study and from literature.**

| **Authors** | **No. Cases** | **Type of seizures** |
| --- | --- | --- |
| *Mikati et al. 2000* | 8/44 (19%) | 4 GTCS; 3 FCS; 1 GMS |
| *Sweney et al. 2009* | 44/103 (43%) | 44 GTCS |
| *Saito et al. 2010* | 1 | ES |
| *Rosewich et al. 2014* | 4/9 | 4 SE |
| *Uchitel et al. 2019* | 51 | 32 (62%) FS (mainly frontal); 11 (21%) GTCS-MS-Absence; 8 ES |
| Present cases | 2/4 | FS |
|  |  |  |

*Abbreviations: GMS= Generalized Myoclonic Seizures; GTCS= Generalized Tonic or Tonic-Clonic Seizures; ES= Epileptic Seizures; FCS= Focal Clonic Seizures; FS= Focal Seizures; MS=Myoclonic Seizures; SE= Status Epilepticus.*
